# The Influence of an Enriched Sport Program on Children’s Sport Motivation in the School Context: The ESA PROGRAM

**DOI:** 10.3389/fpsyg.2020.601000

**Published:** 2020-10-26

**Authors:** Ambra Gentile, Stefano Boca, Yolanda Demetriou, David Sturm, Simona Pajaujiene, Ilona Judita Zuoziene, Fatma Nese Sahin, Özkan Güler, Manuel Gómez-López, Carla Chicau Borrego, Doris Matosic, Antonino Bianco, Marianna Alesi

**Affiliations:** ^1^Ph.D. Program in Health Promotion and Cognitive Sciences, University of Palermo, Palermo, Italy; ^2^Department of Psychology, Educational Sciences and Human Movement, University of Palermo, Palermo, Italy; ^3^Department of Sport and Health Sciences, Technical University of Munich, Munich, Germany; ^4^Department of Coaching Science, Lithuanian Sports University, Kaunas, Lithuania; ^5^Faculty of Sports Science, Ankara University, Ankara, Turkey; ^6^Department of Physical Activity and Sport, Faculty of Sports Sciences, University of Murcia, Murcia, Spain; ^7^Sport Sciences School of Rio Maior, Polytechnic Institute of Santarém - Research Center in Life Quality, Rio Maior, Portugal; ^8^Faculty of Kinesiology, University of Split, Split, Croatia

**Keywords:** motivation, social support, enriched sport program, physical education, gender difference

## Abstract

**Purpose:**

Besides the evident positive effect on body development, physical activity has proven to boost executive functions, especially if the exercises are enriched with cognitive stimuli. Previous studies have shown that introducing challenging exercises in the physical activity routine can also enhance motivation. Therefore, enriching a physical education program with cognitively challenging exercises may also foster children’s motivation during physical education classes, where the motivation is high at the beginning of the school year and low at the end of it. Therefore, the purpose of this paper is to test if a sport program enriched by cognitive stimuli may improve kids’ motivation or take them out from a state of amotivation along the school year.

**Methods:**

A sample of 342 school children (203 boys, 139 girls) took part in the study. Participants were asked to complete a battery of motivation and perceived social support questionnaires before and after they completed the ESA Program, a sport program enriched with cognitive stimuli. Moreover, parents of these children attended four seminars about the importance of supporting children for the practice of regular physical activity (PA). A control group consisting of children that attended the ordinary physical education school class was also included.

**Results:**

A repeated measures MANOVA model showed that the ESA Program was able to improve children’s general motivation, in particular the intrinsic motivation. The program was not effective in social support, but, independently from the group, the family social support in sports activities decreased for females.

**Conclusion:**

Apart from cognitive improvement, the ESA Program can have beneficial effects on children’s sports motivation in physical education, but not on perceived social support.

## Introduction

The practice of sport activities has undoubtedly positive effects on children’s physical fitness and psychological well-being. These benefits concern improved physical fitness, balance, and endurance jump as well as the decrease of obesity and type 2 diabetes (Janssen and LeBlanc, 2010; Golubović et al., 2012; Reid et al., 2015; Navarro-Patón et al., 2019). From the psychological perspective, positive effects concern the increase of self-determination, self-esteem, and self-efficacy, and the decrease of anxiety and depression. Recent studies confirmed its beneficial effects on cognitive functioning that, in turn, is connected to an improvement in academic achievement (Donnelly and Lambourne, 2011; Misuraca et al., 2017; Egger et al., 2019; Harveson et al., 2019; Mavilidi et al., 2020). However, children are likely to drop-out from taking part in sport activities, especially those characterized by parental inactivity (Silva et al., 2019), and it seems that girls’ participation declines more than their males counterparts (Malina, 2001). As showed by the latest Eurobarometer on sport and physical activity (PA), the rates of frequency and levels of engagement in sport or other PA decreases from Northern to Southern countries, and the rate of drop-outs increase with age. Therefore, identifying those factors that prevent children (especially girls’) drop-out can be useful to empower their cognitive functioning and to prevent poor health and obesity.

A review of Sallis et al. (2000) reported a strong relationship across studies between social support by parents and PA. Parents, or caretakers, are considered one of the first sources influence on youth sport-related behavior since they serve as a model and guide for health-enhancing and health-compromising habits (Beets et al., 2010). Apart from family, other studies confirmed that social support coming from friends and school might predict the likelihood to engage in PA (Duncan et al., 2005; Hohepa et al., 2007). Indeed, friends’ social support could have an even stronger impact than parents’ support on children’s likelihood to PA (Efrat, 2009; Loucaides and Tsangaridou, 2017). Moreover, school is an ideal, accessible and cost-effective context to implement interventions aimed at enhancing engagement in PA because of its possibility to involve all cohorts of children and adolescents and its wide application (Piercy et al., 2015). School-based PA interventions concern several domains ranging from specific PE curriculum to classroom activity breaks or after-school programs (Gråstén, 2017).

A recent systematic review analyzed the effects of school-based PA interventions, such as PA components during school lessons or during morning, lunch and afternoon breaks, on a variety of motivational measures of PA in school-aged children and adolescents (Demetriou et al., 2019). Results provided evidences of the efficacy of strategies implemented in the school setting to enhance and maintain students’ motivation toward PA and hence to increase their PA during school and after-school hours.

Nevertheless, children’s engagement in physical education class depends on their motivation (Ntoumanis, 2005). In particular, an adaptive motivational disposition based on positive self-esteem and perception of competence, effort attribution style, task-oriented goals and persistence when faced with failures, increases the probabilities of sport participation and success (Granero-Gallegos et al., 2017). This topic has been framed within the self-determination theory (SDT) by Deci and Ryan (2008). The theory differentiates among three types of self-determined motivation, namely, intrinsic motivation, extrinsic motivation, and amotivation (Ntoumanis, 2005). Intrinsically motivated individuals perform certain behaviors due to interest and joy; this represents the highest self-determined motivation. When individuals engage in an activity because of its outcome (e.g., rewards, praise), they are extrinsically motivated. In physical education, three types of extrinsic motivation have been detected (Goudas et al., 1994), that are identified regulation, introjected regulation, and external regulation. Identified regulation concerns the activity that individuals perform since they previously internalized the value, but they do not necessarily enjoy it. A behavior driven by introjected regulation was slightly internalized, and it is performed with a sense of guilt or shame. The external regulation guides the individuals in the engagement of behaviors directed to attain tangible rewards or avoid punishment. Finally, amotivation represents the absence of motivation. Individuals, thus, display no intention to engage in an activity. However, as noted by Gröpel et al. (2016), when talking about PA, an important feature of motivation is achievement. The authors found that an implicit need for achievement predicts regular engagement in sport activities.

To what concerns sport programs created for children, according to Diamond (2012) the forecasted PA should challenge children throughout the program. Indeed, if kids are not pushed to perform better, they stop improving. Moreover, if the children perceive no challenge, they get bored and abandon the program. Therefore, creating a program stimulating cognitive functions that also challenge participants’ competence may improve children’s cognitive function and, at the same time, sustain their sport motivation (Ryan et al., 1997). However, no studies were found about the effects of a sport program enriched through cognitive stimuli on children’s sport motivation.

The current study hypothesizes a positive effect of an enriched school-based sport program, the ESA Program, on children’s motivation and social support. Enriched Sports Activity program (ESA program) is a sport program enriched through cognitive stimuli, experimented within the Erasmus + Project Enriched Sports Activity Program (ESA Program; Agreement Nr.: Sport-579661-EPP-1-2016-2-IT-SPO-SCP). The project aimed to enhance social inclusion, equal opportunity, and psycho-social well-being in children by stimulating their cognitive growth and motivational aspects (Alesi et al., 2017). The program consisted of physical exercises that were modified to stimulate the three core executive functions, namely inhibitory control, working memory, and task shifting. ESA Program revealed to have positive effects on children’s physical performance (Thomas et al., 2020) and executive functions (in particular, on working memory and task shifting) (Gentile et al., 2020).

The ESA Program session differ from a traditional physical education class in the standardization of the sessions, consisting in a warm-up phase and a stimulation phase, while a physical education class follows less rigid schemes. Moreover, children are encouraged to enhance their skills by increasing the exercise difficulty step-by-step. Finally, a cognitively engaging physical activity produces improvements in executive functions, while mere aerobic exercise does not produce the same enhancement (Schmidt et al., 2016).

In the first phase (t1), data collection was conducted at the beginning of the school year, when children usually show high motivation, while the second data collection (t2) occurred at the end of the school year, when motivation is notably lower compared to the beginning. Therefore, we expect an overall decline in terms of motivation, and children attending the ESA Program should experience less reduction of motivation at t2 compared to those children who did not attend the program, showing a protective effect of the program on motivational decline. Concerning social support, since parents whose children attended ESA Program were also involved in seminars and informative days, we expect a general improvement of perceived social support along the school year.

Finally, we will look for gender differences in motivation. In principle, it is expected a lower degree of internal and identified motivation and a higher degree in amotivation and external regulation in females, since sport is generally considered more suitable for men than for women (Gentile et al., 2018). The same phenomenon should occur for social support from families and friends. If the sports is commonly seen as not suitable for women, then female participants will not receive as much support as the male counterpart.

## Materials and Methods

### Participants

The sample consisted of 342 schoolchildren (203 boys, 139 girls) coming from Italy, Germany, and Lithuania (Table 1). Data were collected in November (t_1_) and in May (t_2_) of the same school year. The study was implemented according to the Helsinki Declaration (Hong Kong revision, September 1989) and received permission from the Lithuanian Sports University’s Research Ethics Committee in Social Sciences with approval No 579661-EPP-1-2016-2-IT-SPO-SCP (2018-02-05).

**TABLE 1:** Participants per country distinguished in Control and Intervention groups.

	Intervention	Control
Italy	77	87
Lithuania	56	37
Germany	38	36
Total	171	160

### Procedure

After parents’ signature of the consent form, children school classes were split in experimental and control group. Children from both experimental (ESA group) and control group completed two forms detecting sport motivation (Lonsdale et al., 2008; Viladrich et al., 2013) and social support (Sallis et al., 2002; Dishman et al., 2010). After 6 months, children from both experimental and control groups completed the same forms for the second time. All the measures were translated and adapted to each context with the authors’ permission.

### Youth Behavioral Regulation in Sport Questionnaire (YBRSQ)

The Youth Behavioral Regulation in Sport Questionnaire (YBRSQ) is an adaptation by Viladrich et al. (2013) of the Behavioral Regulation in Sport Questionnaire (BRSQ), and it is mainly focused on children’s motivation (Lonsdale et al., 2008). The questionnaire is based on Self-Determination Theory (Deci and Ryan, 2008), and consists of 20 items on a 5-point Likert scale (from 1 = strongly disagree to 5 = strongly agree). The questionnaire measures general motivation (4 items, sample item “I participate in my sport because I like it”), amotivation (4 items, sample item “I participate in my sport… but I question why I continue”), external (4 items, sample item “I participate in my sport because people push me to play”), introjected (4 items, sample item “I participate in my sport because I would feel guilty if I quit”) and identified (4 items, sample item “I participate in my sport because the benefits of sports are important to me”) regulations.

### Social Support

Social support was assessed through the adaptation of the Social Support subscale from the Social Provisions Scale (Motl et al., 2004) made by Dishman et al. (2010). The scale is made up of 7 items, detecting perceived social support from family (4 items, sample item “during a typical week, how often has a member of your household [for example, your father, mother, brother, sister, grandparent, or other relative] done a physical activity or played sports with you?”) and from friends (3 items, sample item “during a typical week, how often do your friends encourage you to do physical activities or play sports?”) on a 5-point Likert scale, from 1 = “never” to 5 = “every day.”

### ESA Program

ESA Program is a sport program conceived for children from 7 to 14 years, attending the physical education class in schools. The program’s strengths are the standardization of the warm-up phase and its enrichment with cognitive stimuli, attaining the three core executive functions, namely, inhibition, working memory, and task shifting. The implementation of the program lasted 14 weeks, running twice a week, for a total amount of 27 units. All the units lasted 25 min and were divided into a baseline phase and a stimulation phase. For the baseline phase, children were asked to perform an exercise, while in the stimulation phase, children were asked to follow some specific rules. The forecasted exercises were distinguished for cognitive stimulation and movement domain. The cognitive stimulation concerned the alternate enrichment of the activity with cognitive features that could involve inhibitory control, working memory and task switching stimuli. The inhibitory control stimulation consisted of replicating a gesture, previously associated, and after the coach command, in the execution of another movement, before explained by the coach. For example, when the coach said “skip-ahead,” children had to perform the “kick-ahead” movement. The working memory stimulation occurred through the introduction of a series of exercises that children had to replicate in a reverse order. Finally, regarding task shifting stimulation, children created a circle of exercise, each of them performing one exercise. At the coach’s whistle, they had to change the exercise with the one that the kid ahead was performing. In this way, all the children performed one by one all the exercise of the circuit.

The movement domain could relate to athletic drills, sport ball and smart circuits. Therefore, the program was articulated as follows: the first nine units were classified as beginner level (B), and alternatively concerned athletic drill, sport ball and smart circuits, alternatively enriched through inhibitory control, working memory and task shifting stimulation. An identical structure was replicated for the Intermediate level (I) and for the advanced level (A). The standardization of the protocol across the European Countries was guaranteed by a video-tutorial uploaded on an Internet platform. The ESA Program was implemented ensuring a safe environment for children.

### Parents Involved in ESA Program

Parents whose children attended the ESA Program were involved in four seminars of 1 h each about the benefits of PA in children. Specifically, during the first seminar, the main objectives of the ESA Program were introduced. Moreover, parents participated in a group discussion about the sport motivation of their children. The second seminar focused on raising awareness about the importance of regular PA in children with a final group discussion on the topic. During the third seminar, parents were informed about the harmful effects of physical inactivity during adulthood and the benefits of PA in terms of cognitive functioning. The fourth and last seminar focused on the identification of parental support mode and support for children’s autonomy.

### Data Analysis

First, descriptive statistics were calculated on the sample (Table 2). A repeated measures Multivariate Analysis of Variance (MANOVA) with Group (Experimental vs. Control), Time (pre-test vs. post-test condition), Gender (male vs. female), and Motivation (Intrinsic, Integrated, Introjected, Extrinsic, Amotivation) was calculated to detect the general effect of ESA Program on motivational aspects, and to test whether any gender differences exist. The model was designed following the procedure described by O’Brien and Kaiser (1985). Afterward, a *post-hoc* analysis with repeated measure ANOVA was performed to detect which aspects of motivation were affected the most. Concerning social support, since family and peers promote PA independently from one another, two distinct repeated measure ANOVA with Group (Experimental vs. Control), Time (pre-test vs. post-test condition), and Gender (male vs. female) were conducted to detect the effect of ESA Program on perceived social support and test for gender differences.

**TABLE 2:** Mean and SD of intervention and control groups in pre- and post-test condition.

	Intervention				Control			
	Pre-test		Post-test		Pre-test		Post-test	
	*M*	*SD*	*M*	*SD*	*M*	*SD*	*M*	*SD*
Intrinsic regulation	17.70	3.02	17.30	3.47	17.60	3.13	16.30	4.64
Identified regulation	16.30	3.20	14.10	5.95	16.0	3.35	14.80	4.71
Introjected regulation	9.14	4.42	9.98	4.23	9.11	3.88	10.50	4.32
External regulation	7.82	3.82	8.43	3.50	7.41	3.51	8.63	3.79
Amotivation	7.94	3.45	9.63	3.94	7.96	3.55	10.20	4.25
Social support from family	13.60	3.17	12.90	3.73	12.10	4.10	12.40	4.16
Social support from friends	7.99	3.11	8.38	3.34	8.14	3.26	8.32	3.26

## Results

### The Effect of ESA Program on Children’s Motivation

The repeated measure MANOVA revealed a violation of the sphericity assumption (*W* = 0.71, *p* < 0.001). Therefore, Wilks Lambda (Λ) was chosen as estimator of the test statistics for multivariate effect. First of all, gender did not produce any significant main effect and was hence excluded from subsequent analyses. The model thus became a 2 (intervention vs. control) × 2 (pre-test vs. post-test) × 5 (intrinsic, introjected, identified, external, amotivation) design, with the first factor manipulated between participants. An interaction effect between Time x Motivation was detected [Λ = 0.75, *F*_(4, 291)_ = 24.42, *p* < 0.001] meaning that motivation has changed over time. A significant three-way interaction between Time, Motivation and Group emerged [Λ = 0.96, *F*_(4, 291)_ = 2.70, *p* = 0.031] meaning that the evolution of motivation over time depends on the participation into the ESA program.

**FIGURE 1:**
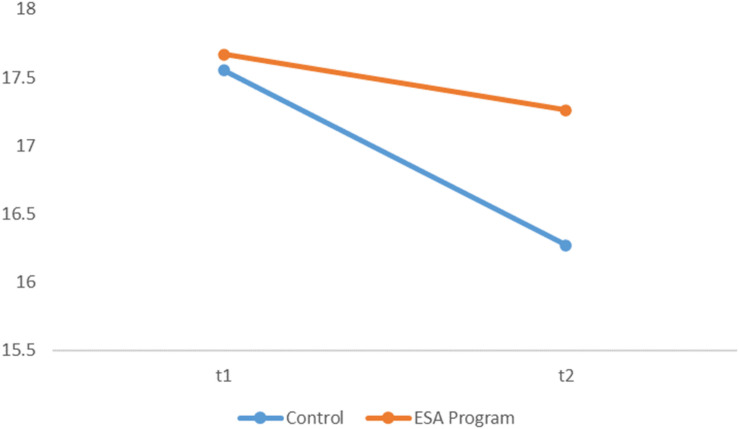
Intrinsic motivation for ESA Program and control group.

The repeated measure ANOVA revealed that ESA Program had a protective effect on intrinsic motivation [*F*_(1, 294)_ = 4.15, *p* = 0.04; *d* = 0.28; Figure 1], but not on identified regulation [*F*_(1, 304)_ = 2.77, *p* = 0.09; *d* = −0.31), introjected regulation [*F*_(1, 294)_ = 0.98, *p* = 0.32; *d* = −0.13), external regulation [*F*_(1, 294)_ = 1.99, *p* = 0.16; *d* = −0.17], and amotivation [*F*_(1, 294)_ = 1.48, *p* = 0.22; *d* = −0.17]. Intrinsic motivation tends to decrease along the school year but only for children who did not take part in the ESA Program.

### The Effect of ESA Program on Children Social Support

**FIGURE 2:**
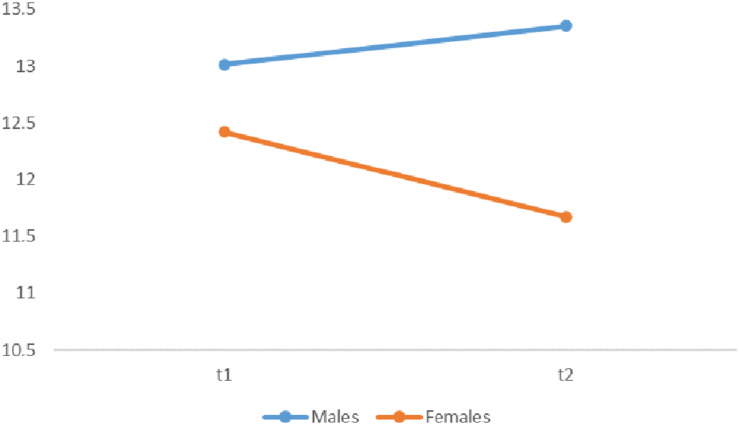
Gender differences in perceived social support from family.

Concerning perceived social support from family, a significant effect of gender was observed [*F*_(1, 289)_ = 6.00, *p* = 0.015, η*_*p*_* = 0.02]. Specifically, girls perceive less social support in sports activity from family than boys, independently from the participation to the ESA Program. Moreover, an interaction between Gender × Time emerged [*F*_(1, 289)_ = 7.60, *p* = 0.006, η*_*p*_* = 0.03; see Table 3] indicating that the evolution of perceived support from family over the school year varies differently for boys and girls (mean difference_*boys*_ = 0.40, mean difference_*girls*_ = −0.70, *d* = 0.29; Figure 2). Regarding the ESA Program, no interaction Group × Time was found [*F*_(1, 291)_ = 3.00, *p* = 0.09], and the program effect did not show gender differences [*F*_(1, 289)_ = 0.51, *p* = 0.47].

**TABLE 3:** Mean and SD distinguished by gender between pre- and post-test.

	Males				Females			
	Pre-test		Post-test		Pre-test		Post-test	
	*M*	*SD*	*M*	*SD*	*M*	*SD*	*M*	*SD*
Social support from family	13.0	3.61	13.4	3.49	12.40	3.94	11.7	4.37
Social support from friends	8.37	3.19	8.85	3.24	7.57	3.11	7.69	3.26

Concerning perceived social support from friends, a significant effect of gender emerged [*F*_(1, 287)_ = 12.86, *p* < 0.001, η*_*p*_* = 0.043], where girls perceive less support from friends than boys. The ESA Program did not produce any significant main effect [*F*_(1, 287)_ = 0.44, *p* = 0.50, η*_*p*_* = 0.002] nor interactions with time [*F*_(1, 287)_ = 0.53, *p* = 0.46, η*_*p*_* = 0.002] and gender [*F*_(1, 287)_ = 0.02, *p* = 0.86, η*_*p*_* = 0.000].

## Discussion

Keeping children’s sport motivation high and supporting them throughout the sport practice can prevent their drop-out from the sport practice in the future. Within this perspective, the current paper aimed at assessing if a school-based sport program enriched by cognitive challenges could improve children’s sport motivation or, at least, limit motivational loss over the school year. From the analyses, we found that ESA Program has a general effect on children’s motivation in both boys and girls, compared to common physical education class. In particular, the program seems to have a protecting effect on children’s intrinsic motivational decline along the school year (Archambault et al., 2010).

Previous research has shown that intrinsic motivation is the main determinant of the engagement in PA over time (Richard et al., 1997). It’s largely recognized how children that are motivated to PA from external sources and with controlled forms of motivation are more likely to perform PA with a sense of pressure and coercion by effecting, in the long term, on decreasing the enjoyment and, in a complementary way, increasing negative affect and attitudes toward PA (Costa et al., 2020). Thus, the introduction of cognitive challenging exercises enhances intrinsic motivation, and hence favors the adherence to sport programs, avoiding the drop out. Naturally, task difficulties must be adapted to the participants’ skill levels (Mandigo and Holt, 2000). In sum, the analysis of motivational data revealed that ESA Program is a valid tool for maintaining a high level of intrinsic motivation in children involved in sport activities.

Considering that parents involved in the program attended four seminars about the importance of supporting children in the practice of PA, we expected an enhancement in perceived social support from family in participants involved in the ESA program. Unexpectedly, ESA Program did not influence children perceived social support from family and friends. Perceived social support from family declined in girls over the school year. The same did not happen for boys who kept perceiving a support from their families all along the year. This result is in line with Anderssen and Wold (1992), but appears to be in contrast to Davison (2004), who found no gender differences in parents’ support to the adolescents’ activity.

Social support coming from family is one of the strongest predictors of motivation and children’s future participation in sports (Beets et al., 2010). The differences in perceived social support from family could explain the higher rate of girls’ drop-out from the sport practice compared to boys. These gender differences do not emerge by chance. They are probably related to the persistence of gender stereotypes in sports which leads to considering these practices as unnecessary activities for girls (Eccles and Harold, 1991; Gentile et al., 2018). Moreover, regardless of the participation in the program, perceived social support of friends revealed to be stable both for males and females.

In addition to the enrichment with challenging cognitive tasks, the strength point of this research is the delivery of ESA program in school context. Children spend a large amount of their day hours in school, so this is a perfect context to address all cohorts of students over prolonged periods of time. School revealed to be an increasing setting to implement accessible and effective programs to improve motivation to PA (Wallhead et al., 2014).

Although the results of this study are encouraging, certain theoretical and methodological shortcomings must be acknowledged. First of all, we did not control for some variables that might influence the study outcome, such as socioeconomic status or motivational climate. Moreover, we did not control for the perception of self-competence and self-efficacy. The role of these variables on the intention to go on taking part in sport activities and sport programs will be investigated in future research. Finally, Germany did not carry out all the units the program, and spread some leaflet about the importance of sports activity instead of conducting seminars with parents. However, the results are encouraging even if the program was not entirely conducted there.

Another relevant condition to control in the future is the relationship between the intensity of PA and the strength of the motivation. Ekkekakis et al. (2011) found that sub-threshold intensity exercises lasting from 10 to 30 min produce a pleasant affective changes for most individuals. The matching among physical activity task intensity or difficulty and individual skills needs to be carefully considered to establish challenges suitable for each individual and, as a consequence, stimulate positive affect such as enjoyment, pride, and adaptive motivation. As concern methodological limitations, all variables were measured through self-report questionnaires. This procedure allow respondent to systematically manage their answers to show to the interviewer a positive self-image. For the psychological constructs considered there are alternative assessment tool available relying on implicit measures (Lawrence and Jordan, 2009). Nevertheless, these assessment methods are complicated, time consuming, and require standardized lab equipment. For these reasons they are rarely used in large scale investigations like this one.

From a psycho-educational perspective, practitioners and families must be aware of the key role of PA to promote well-being and to impact on the whole child’s development. It is important that public health organizations address the primary aim to plan and implement evidence-based school programs to encourage sport motivation and the enjoyment in sport and PA practice. The results of the current study could be useful for physical education teachers in structuring classes able to foster children’s sport motivation. Moreover, school teachers should be aware of the gender differences in social support, for avoiding girls’ drop-out during adolescence.

## Data Availability Statement

The raw data supporting the conclusions of this article will be made available by the authors, without undue reservation, to any qualified researcher.

## Ethics Statement

The studies involving human participants were reviewed and approved by the Lithuanian Sports University’s Research Ethics Committee in Social Sciences with approval no. 579661-EPP-1-2016-2-IT-SPO-SCP (2018-02-05). Written informed consent to participate in this study was provided by the participants’ legal guardian/next of kin.

## Author Contributions

AG, AB, and MA: conceptualization. AG, AB, and SB: data curation and formal analysis. AB and MA: funding acquisition and project administration. FS, ÖG, SP, YD, DS, MG-L, and IZ: investigation. AB, SB, and MA: methodology. DM, CB, IZ, and OD: resources. FS and DS: software. YD, SP, CB, and ÖG: supervision. MG-L and IZ: validation. AG and MA: visualization. AG, SB, AB, MA, and MG-L: roles/writing – original draft. FS, IZ, SP, YD, DS, CB, and ÖG: writing – review and editing. All authors contributed to the article and approved the submitted version.

## Conflict of Interest

The authors declare that the research was conducted in the absence of any commercial or financial relationships that could be construed as a potential conflict of interest.

## References

[B1] Alesi M. Silva C. Borrego C. Monteiro D. Genchi R. Polizzi V. (2017). Cognitive and motivational monitoring during enriched sport activities in a sample of children living in Europe. The Esa Program. *J. Funct. Morphol. Kinesiol.* 2:46 10.3390/jfmk2040046

[B2] Anderssen N. Wold B. (1992). Parental and peer influences on leisure-time physical activity in young adolescents. *Res. Quart. Exer. Sport* 63, 341–348. 10.1080/02701367.1992.10608754 1439157

[B3] Archambault I. Eccles J. S. Vida M. N. (2010). Ability self-concepts and subjective value in literacy: joint trajectories from grades 1 through 12. *J. Educ. Psychol.* 102 804–816. 10.1037/a0021075

[B4] Beets M. W. Cardinal B. J. Alderman B. L. (2010). Parental social support and the physical activity-related behaviors of youth: a review. *Health Educ. Behav.* 37 621–644. 10.1177/1090198110363884 20729347

[B5] Costa S. Bianco A. Polizzi V. Alesi M. (2020). Happiness in physical activity: a longitudinal examination of children motivation and negative affect in physical activity. *J. Happ. Stud.* 0 1–13.

[B6] Davison K. K. (2004). Activity-related support from parents, peers, and siblings and adolescents’ physical activity: are there gender differences? *J. Phys. Activ. Health* 1 363–376. 10.1123/jpah.1.4.363

[B7] Deci E. L. Ryan R. M. (2008). Self-determination theory: a macrotheory of human motivation, development, and health. *Can. Psychol.* 49 182–185. 10.1037/a0012801

[B8] Demetriou Y. Reimers A. K. Alesi M. Scifo L. Borrego C. C. Monteiro D. (2019). Effects of school-based interventions on motivation towards physical activity in children and adolescents: protocol for a systematic review. *Syst. Rev.* 8:113. 10.1186/s13643-019-1029-1 PMC6511217 31077254

[B9] Diamond A. (2012). Activities and programs that improve children’s executive functions. *Curr. Direct. Psychol. Sci.* 21 335–341. 10.1177/0963721412453722 25328287 PMC4200392

[B10] Dishman R. K. Hales D. P. Sallis J. F. Saunders R. Dunn A. L. Bedimo-Rung A. L. (2010). Validity of social-cognitive measures for physical activity in middle-school girls. *J. Pediatr. Psychol.* 35 72–88. 10.1093/jpepsy/jsp031 19433571 PMC2910934

[B11] Donnelly J. E. Lambourne K. (2011). Classroom-based physical activity, cognition, and academic achievement. *Prev. Med.* 52 S36–S42. 21281666 10.1016/j.ypmed.2011.01.021

[B12] Duncan S. C. Duncan T. E. Strycker L. A. (2005). Sources and types of social support in youth physical activity. *Health Psychol.* 24 3–10. 10.1037/0278-6133.24.1.3 15631557

[B13] Eccles J. S. Harold R. D. (1991). Gender differences in sport involvement: applying the Eccles’ expectancy-value model. *J. Appl. Sport Psychol.* 3 7–35. 10.1080/10413209108406432

[B14] Efrat M. (2009). Relationship between Peer and/or friends’ influence and physical activity among elementary school children. *Calif. J. Health Promot.* 7 48–61. 10.32398/cjhp.v7isi.2000

[B15] Egger F. Benzing V. Conzelmann A. Schmidt M. (2019). Boost your brain, while having a break! The effects of long-term cognitively engaging physical activity breaks on children’s executive functions and academic achievement. *PLoS One* 14:e0212482. 10.1371/journal.pone.0212482 30840640 PMC6402646

[B16] Ekkekakis P. Parfitt G. Petruzzello S. J. (2011). The pleasure and displeasure people feel when they exercise at different intensities. *Sports medicine* 41 641–671. 10.2165/11590680-000000000-00000 21780850

[B17] Gentile A. Boca S. Giammusso I. (2018). ‘You play like a Woman!’effects of gender stereotype threat on women’s performance in physical and sport activities: a meta-analysis. *Psychol. Sport Exerc.* 39 95–103. 10.1016/j.psychsport.2018.07.013

[B18] Gentile A. Boca S. Şahin F. N. Güler Ö Pajaujiene S. Indriuniene V. (2020). The effect of an enriched sport program on children’s executive functions: the ESA Program. *Front. Psychol.* 11:657. 10.3389/fpsyg.2020.00657 32411039 PMC7198739

[B19] Golubović Š Maksimović J. Golubović B. Glumbić N. (2012). Effects of exercise on physical fitness in children with intellectual disability. *Res. Dev. Disabil.* 33 608–614. 10.1016/j.ridd.2011.11.003 22155534

[B20] Goudas M. Biddle S. Fox K. (1994). Achievement goal orientations and intrinsic motivation in physical fitness testing with children. *Pediatr. Exerc. Sci.* 6 159–167. 10.1123/pes.6.2.159

[B21] Granero-Gallegos A. Gómez-López M. Rodríguez-Suárez N. Abraldes J. A. Alesi M. Bianco A. (2017). Importance of the motivational climate in goal, enjoyment, and the causes of success in handball players. *Front. Psychol.* 8:2081. 10.3389/fpsyg.2017.02081 29250011 PMC5717397

[B22] Gråstén A. (2017). School-based physical activity interventions for children and youth: keys for success. *J. Sport Health Sci.* 6 290–291. 10.1016/j.jshs.2017.03.001 30356591 PMC6189019

[B23] Gröpel P. Wegner M. Schüler J. (2016). Achievement motive and sport participation. *Psychol. Sport Exerc.* 27 93–100. 10.1016/j.psychsport.2016.08.007

[B24] Harveson A. T. Hannon J. C. Brusseau T. A. Podlog L. Papadopoulos C. Hall M. S. (2019). Acute exercise and academic achievement in middle school students. *Int. J. Environ. Res. Publ. Health* 16:3527. 10.3390/ijerph16193527 PMC6801915 31547214

[B25] Hohepa M. Scragg R. Schofield G. Kolt G. S. Schaaf D. (2007). Social support for youth physical activity: importance of siblings, parents, friends and school support across a segmented school day. *Int. J. Behav. Nutr. Phys. Activ.* 4:54. 10.1186/1479-5868-4-54 17996070 PMC2186357

[B26] Janssen I. LeBlanc A. G. (2010). Systematic review of the health benefits of physical activity and fitness in school-aged children and youth. *Int. J. Behav. Nutr. Phys. Activ.* 7:40. 10.1186/1479-5868-7-40 20459784 PMC2885312

[B27] Lawrence S. Jordan P. (2009). Testing an explicit and implicit measure of motivation. *Int. J. Organ. Anal.* 17 103–120. 10.1108/19348830910948959

[B28] Lonsdale C. Hodge K. Rose E. A. (2008). The behavioral regulation in sport questionnaire (BRSQ): instrument development and initial validity evidence. *J. Sport Exerc. Psychol.* 30 323–355. 10.1123/jsep.30.3.323 18648109

[B29] Loucaides C. A. Tsangaridou N. (2017). Associations between parental and friend social support and children’s physical activity and time spent outside playing. *Int. J. Pediatr.* 2017:7582398. 10.1155/2017/7582398 PMC5350297 28348605

[B30] Malina R. M. (2001). Physical activity and fitness: pathways from childhood to adulthood. *Am. J. Hum. Biol.* 13 162–172. 10.1002/1520-6300(200102/03)13:2<162::aid-ajhb1025>3.0.co;2-t 11460860

[B31] Mandigo J. L. Holt N. L. (2000). Putting theory into practice: how cognitive evaluation theory can help us motivate children in physical activity environments. *J. Phys. Educ. Recreat. Dance* 71 44–49. 10.1080/07303084.2000.10605984

[B32] Mavilidi M. F. Drew R. Morgan P. J. Lubans D. R. Schmidt M. Riley N. (2020). Effects of different types of classroom physical activity breaks on children’s on-task behaviour, academic achievement and cognition. *Acta Paediatr.* 109 158–165. 10.1111/apa.14892 31168863

[B33] Misuraca R. Miceli S. Teuscher U. (2017). Three effective ways to nurture our brain. *Eur. Psychol.* 22 101–120. 10.1027/1016-9040/a000284

[B34] Motl R. W. Birnbaum A. S. Kubik M. Y. Dishman R. K. (2004). Naturally occurring changes in physical activity are inversely related to depressive symptoms during early adolescence. *Psychosom. Med.* 66 336–342. 10.1097/01.psy.0000126205.35683.0a 15184692

[B35] Navarro-Patón R. Lago-Ballesteros J. Basanta-Camiño S. Arufe-Giraldez V. (2019). Relation between motivation and enjoyment in physical education classes in children from 10 to 12 years old. *J. Hum. Sport Exerc.* 14 527–537.

[B36] Ntoumanis N. (2005). A prospective study of participation in optional school physical education using a self-determination theory framework. *J. Educ. Psychol.* 97 444–453. 10.1037/0022-0663.97.3.444

[B37] O’Brien R. G. Kaiser M. K. (1985). MANOVA method for analyzing repeated measures designs: an extensive primer. *Psychol. Bull.* 97 316–333. 10.1037/0033-2909.97.2.316 3983301

[B38] Piercy K. L. Dorn J. M. Fulton J. E. Janz K. F. Lee S. M. McKinnon R. A. (2015). Opportunities for public health to increase physical activity among youths. *Am. J. Public Health* 105 421–426. 10.2105/ajph.2014.302325 25602864 PMC4330821

[B39] Reid M.-A. MacCormack J. Cousins S. Freeman J. G. (2015). Physical activity, school climate, and the emotional health of adolescents: findings from 2010 Canadian Health Behaviour in School-Aged Children (HBSC) study. *Schl. Ment. Health* 7 224–234. 10.1007/s12310-015-9150-3

[B40] Richard M. Christina M. F. Deborah L. S. Rubio N. Kennon M. S. (1997). Intrinsic motivation and exercise adherence. *Int. J. Sport Psychol.* 28 335–354.

[B41] Ryan R. M. Frederick C. M. Lepes D. Rubio N. Kennon M. S. (1997). Intrinsic motivation and exercise adherence. *Int. J. Sport Psychol.* 28 335–354.

[B42] Sallis J. F. Prochaska J. J. Taylor W. C. (2000). A review of correlates of physical activity of children and adolescents. *Med. Sci. Sports Exerc.* 32 963–975. 10.1097/00005768-200005000-00014 10795788

[B43] Sallis J. F. Taylor W. C. Dowda M. Freedson P. S. Pate R. R. (2002). Correlates of vigorous physical activity for children in grades 1 through 12: comparing parent-reported and objectively measured physical activity. *Pediatr. Exerc. Sci.* 14 30–44. 10.1123/pes.14.1.30

[B44] Schmidt M. Benzing V. Kamer M. (2016). Classroom-based physical activity breaks and children’s attention: cognitive engagement works! *Front. Psychol.* 7:1474. 10.3389/fpsyg.2016.01474 27757088 PMC5047899

[B45] Silva D. R. P. Werneck A. O. Collings P. Fernandes R. A. Ronque E. R. V. Sardinha L. B. (2019). Identifying children who are susceptible to dropping out from physical activity and sport: a cross-sectional study. *Sao Paulo Med. J.* 137 329–335. 10.1590/1516-3180.2018.0333050719 31691765 PMC9744009

[B46] Thomas E. Bianco A. Tabacchi G. Marques, da Silva C. Loureiro N. (2020). Effects of a physical activity intervention on physical fitness of schoolchildren: the enriched sport activity program. *Int. J. Environ. Res. Publ. Health* 17:1723. 10.3390/ijerph17051723 32155773 PMC7084442

[B47] Viladrich C. Appleton P. R. Quested E. Duda J. L. Alcaraz S. Heuzé J.-P. (2013). Measurement invariance of the behavioural regulation in sport questionnaire when completed by young athletes across five European countries. *Int. J. Sport Exerc. Psychol.* 11 384–394. 10.1080/1612197x.2013.830434

[B48] Wallhead T. L. Garn A. C. Vidoni C. (2014). Effect of a sport education program on motivation for physical education and leisure-time physical activity. *Res. Q. Exerc. Sport* 85 478–487. 10.1080/02701367.2014.961051 25412130

